# Cardiac MRI assessment of myocardial viability in chronic myocardial infarction: how should we do it?

**DOI:** 10.3389/fcvm.2024.1377230

**Published:** 2024-03-20

**Authors:** Ismail M. Kabakus, Jordan H. Chamberlin, Emily J. Miller

**Affiliations:** Department of Radiology and Radiological Science, Medical University of South Carolina, Charleston, SC, United States

**Keywords:** CMR, MI, CABG, viability, DE

## Introduction

Myocardial infarction (MI) remains a significant global health concern prompting ongoing advancements in diagnostic and therapeutic approaches. Accurate assessment of myocardial viability is crucial in guiding therapeutic decisions and predicting patient outcomes. While the body of literature on cardiac MRI (CMR) provides insights into viability assessment, existing studies lack comprehensive analyses to inform practitioners about the nuances of measurements and their implications for patient selection and outcomes in chronic infarction. This article aims to propose an assessment method and discuss the need for new research.

## Challenge

**Figure 1:**
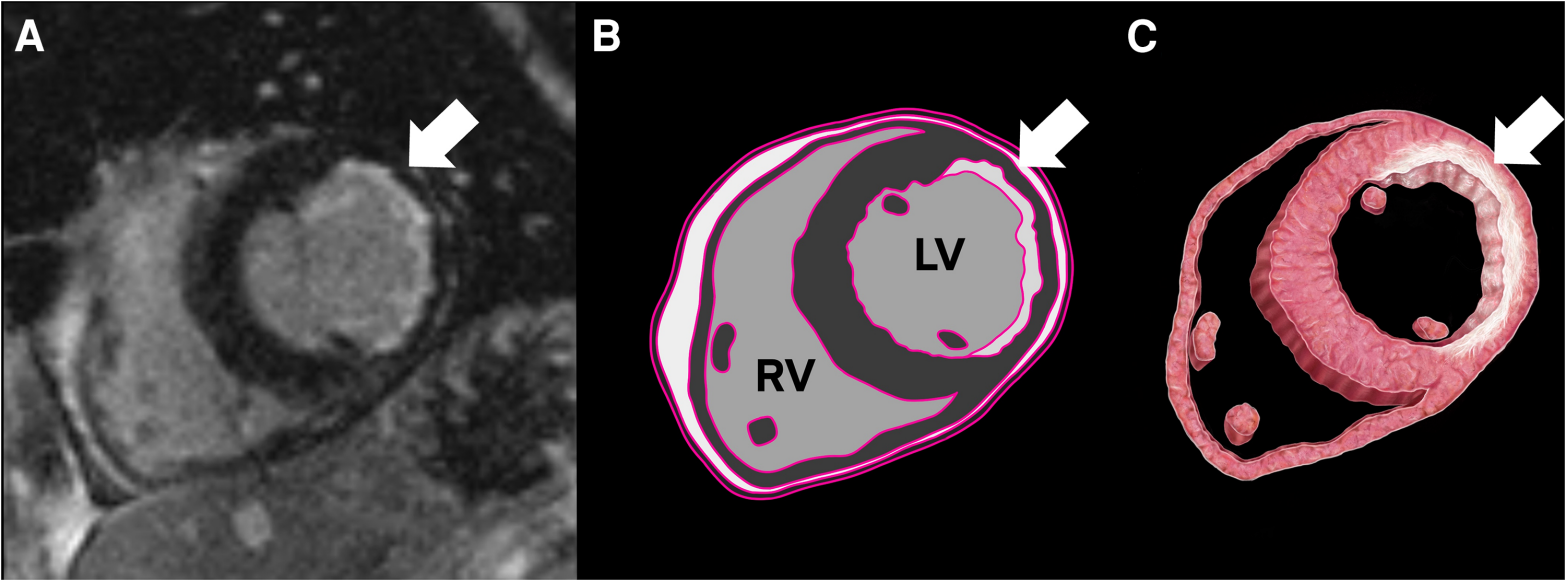
Cardiac MRI (CMR) with a delayed enhancement sequence, revealing subendocardial delayed enhancement and thinning in the lateral wall, consistent with chronic infarct (**A**). The illustrations highlight fibrosis corresponding to the CMR delayed enhancement (**B**,**C**).

The current literature recommends assessing viability by calculating the percentage of non-enhanced myocardium across the full myocardial thickness (1). This approach aids in determining the percentage of the viable myocardium, providing valuable information for risk stratification and surgical candidacy. Although effective in evaluating the transmurality of ischemic/infarct-related changes in cases of acute MI, this method is not suitable for assessing chronic infarction (Figure 1), where the myocardium has already undergone thinning.

## Solution

Assessing myocardial infarct transmurality during the chronic phase requires additional considerations. Following an MI, the affected myocardial region undergoes a complex remodeling process, which includes necrosis of myocardial cells, inflammation, and scar formation, leading to changes in myocardial thickness and function. This remodeling process results in the thinning of the myocardial wall and expansion of the extracellular matrix, indicative of scar tissue formation (2). The chronic phase is characterized by myocardial remodeling, leading to wall thinning in the infarcted region. Accurate estimation of myocardial viability requires determining the initial myocardial thickness, achieved by referencing the healthy myocardium adjacent to the infarcted region. Hence, the authors propose estimating myocardial viability by determining the percentage of non-enhanced myocardium across the neighboring unaffected myocardial thickness adjacent to the infarcted area. This suggestion is supported by the observations that both the degree of wall thinning and transmurality alone are predictive, but not completely specific, for functional recovery of myocardium after revascularization. The authors propose cardiac reserve may be better evaluated by evaluating both as a single entity (3). A similar method was mentioned in another article as an indirect measurement technique; however, the authors did not prefer its use over the conventional method (4).

## Illustrative scenario

Initial healthy myocardial thickness: 10 mm

Total thickness of remodeled myocardium: 5 mm

Delayed enhancement in remodeled myocardium: 2 mm

Non-delayed enhancement in remodeled myocardium: 3 mm

**Figure 2:**
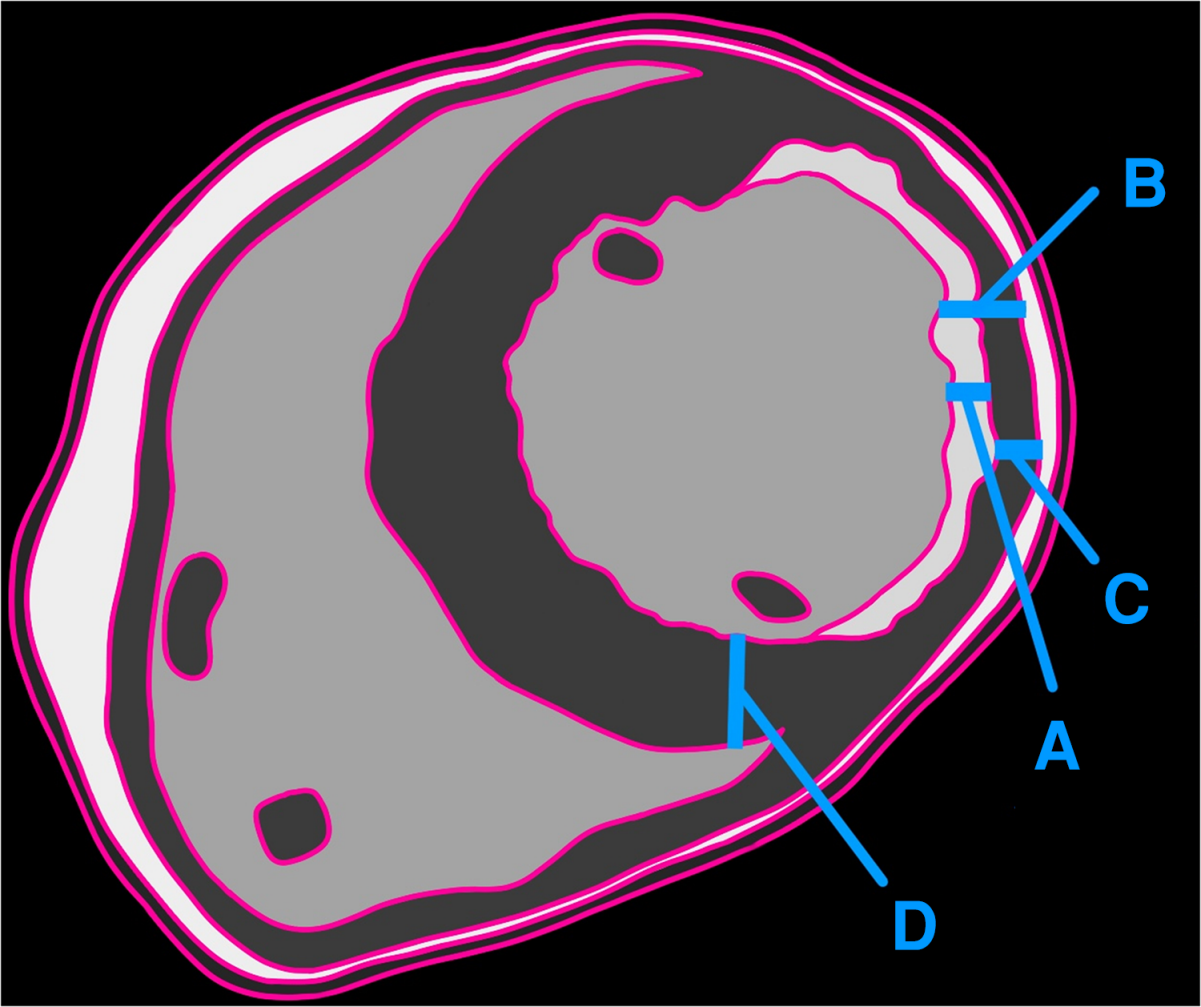
Illustration showing the thickness of delayed enhancement/fibrosis (**A**), the full thickness of the remodeled myocardium (**B**), the thickness of the non-enhancing myocardium (**C**), and the full thickness of the adjacent healthy myocardium (**D**). The authors recommend calculating viability as **C**/**D**, whereas the traditional method employs **C**/**B**.

According to the existing approach, the calculated viable myocardial percentage is 60% (non-delayed enhancement/total thickness of remodeled myocardium) (Figure 2). However, when compared with the adjoining normal myocardium, the viable myocardium is actually 30% (non-delayed enhancement/initial healthy myocardial thickness). This substantial difference in results raises concerns. Given that a 50% threshold is a critical criterion for determining candidacy for coronary artery bypass grafting (CABG) surgery, the conventional method deems revascularization viable, whereas the proposed approach suggests the tissue may not be viable.

## A case report

A 42-year-old patient underwent CMRI for viability assessment. The conventional assessment method yielded a viable myocardial percentage of 53%. In contrast, applying a proposed assessment method resulted in a recalculated viable myocardial percentage of 40% (as illustrated in Supplementary Image S1). Initial diagnostic procedures included an echocardiogram conducted 1 day before the CMRI, which revealed a dilated left ventricle with a significantly reduced ejection fraction of 28%, indicative of impaired systolic function. Following the viability assessment and based on the initial conventional method's findings, the patient underwent CABG. A follow-up echocardiogram post-CABG showed no substantial improvement in the patient's systolic function. The echocardiogram reported a persistently dilated left ventricle with an ejection fraction of 29%.

## Discussion

The rationale of this proposal is akin to the surgeon's knot: are we cutting too long or too short? Is the absolute or the relative viability of myocardial tissue more important to patient prognosis and success in revascularization? As radiologists, are we leaving too many patients on the table—or taking too many—based on imperfect measurements? These questions remain unanswered, and as surgical revascularization remains a mainstay of treatment, there is an opportunity for improved patient selection. Therefore, a more accurate understanding of the extent of myocardial damage during the chronic phase can guide long-term management strategies, including the optimization of medical therapy, timing of interventions, and risk stratification for adverse cardiovascular events. This knowledge will contribute to improving patient outcomes and enhancing the overall quality of care in the post-MI setting.

Future CMR research endeavors should seek to elucidate the optimal method for assessing transmurally/viability in chronic MI. Emerging technologies, such as artificial intelligence, myocardial mapping, and advances in MRI technology, hold promise in enhancing the accuracy of transmurality assessments.

In summary, we propose an innovative approach for evaluating myocardial viability by determining the percentage of non-enhanced myocardium across the neighboring unaffected myocardial thickness adjacent to the infarcted area.

## Supplementary material

Supplementary Image S1 Cardiac MRI (CMR) showing the thickness of delayed enhancement/fibrosis (**A**), the full thickness of the remodeled myocardium (**B**), the thickness of non-enhancing myocardium (**C**), and the full thickness of adjacent healthy myocardium (**D**). The authors recommend calculating viability as [**C**/**D**], while the traditional method employs [**C/B**].
