# Supplementary figures and images for: Cardiac MRI assessment of myocardial viability in chronic myocardial infarction: how should we do it?
Source: Front Cardiovasc Med. 2024 Mar 20;11:1377230. doi: 10.3389/fcvm.2024.1377230 (PMC10989678; doi:10.3389/fcvm.2024.1377230)

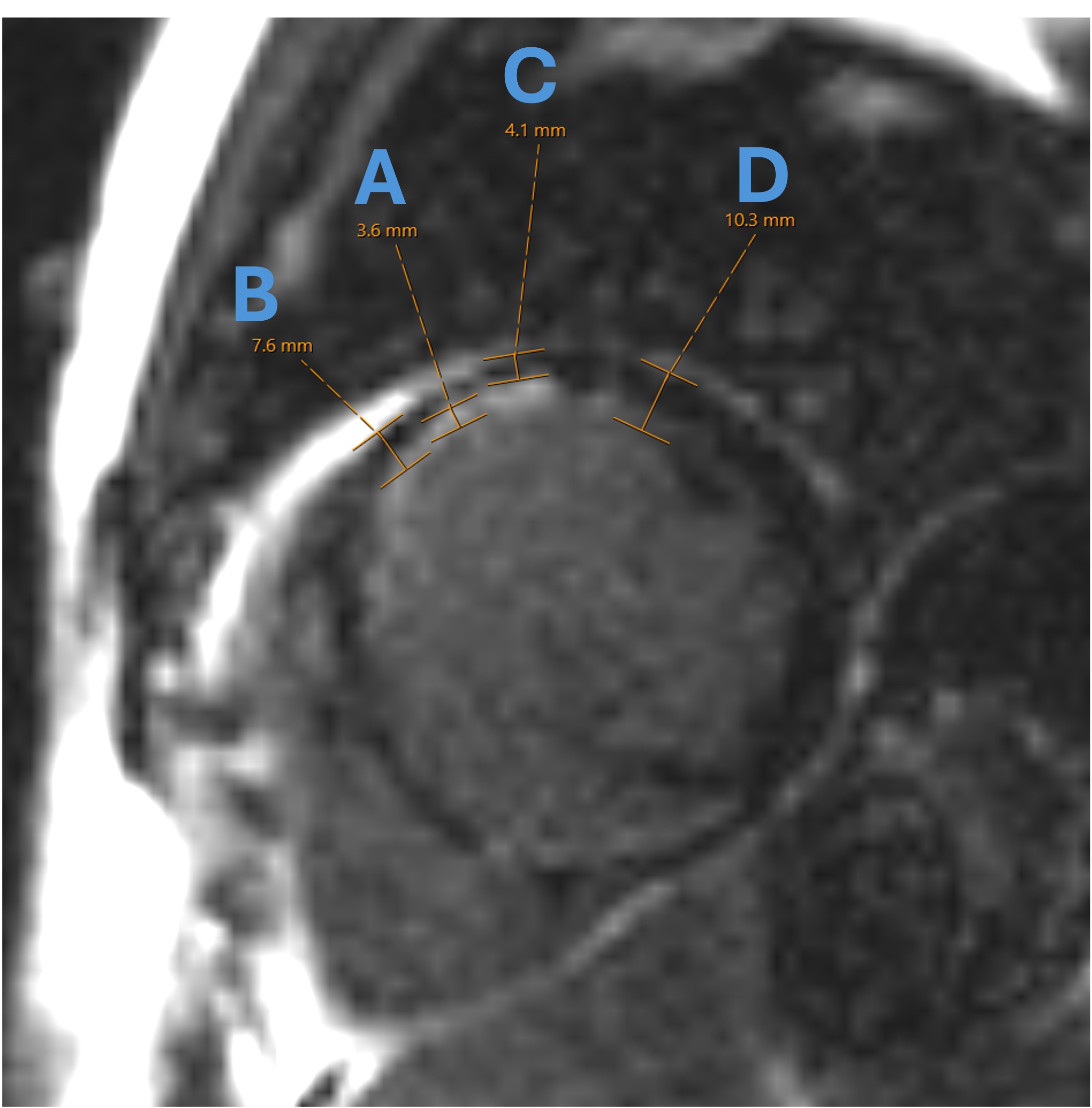

Supplement: Supplementary Image S1 — Cardiac MRI (CMR) showing the thickness of delayed enhancement/fibrosis (A), the full thickness of the remodeled myocardium (B), the thickness of non-enhancing myocardium (C), and the full thickness of adjacent healthy myocardium (D). The authors recommend calculating viability as [C/D], while the traditional method employs [C/B]. [file Image1.jpeg]
